# Ca^2+^ influx-mediated dilation of the endoplasmic reticulum and c-FLIP_L_ downregulation trigger CDDO-Me–induced apoptosis in breast cancer cells

**DOI:** 10.18632/oncotarget.4065

**Published:** 2015-05-25

**Authors:** Soo Ah Jeong, In Young Kim, A Reum Lee, Mi Jin Yoon, Hyeseong Cho, Jong-Soo Lee, Kyeong Sook Choi

**Affiliations:** ^1^ Department of Biochemistry, Ajou University School of Medicine, Suwon, Korea; ^2^ Graduate Program of Cancer Biology, Department of Biomedical Sciences, Ajou University School of Medicine, Suwon, Korea; ^3^ Department of Life Science, Ajou University, Suwon, Korea

**Keywords:** CDDO-Me, endoplasmic reticulum, Ca^2+^, c-FLIP_L_, apoptosis

## Abstract

The synthetic triterpenoid 2-cyano-3, 12-dioxooleana-1, 9(11)-dien-C28-methyl ester (CDDO-Me) is considered a promising anti-tumorigenic compound. In this study, we show that treatment with CDDO-Me induces progressive endoplasmic reticulum (ER)-derived vacuolation in various breast cancer cells and ultimately kills these cells by inducing apoptosis. We found that CDDO-Me–induced increases in intracellular Ca^2+^ levels, reflecting influx from the extracellular milieu, make a critical contribution to ER-derived vacuolation and subsequent cell death. In parallel with increasing Ca^2+^ levels, CDDO-Me markedly increased the generation of reactive oxygen species (ROS). Interestingly, there exists a reciprocal positive-regulatory loop between Ca^2+^ influx and ROS generation that triggers ER stress and ER dilation in response to CDDO-Me. In addition, CDDO-Me rapidly reduced the protein levels of c-FLIP_L_ (cellular FLICE-inhibitory protein) and overexpression of c-FLIP_L_ blocked CDDO-Me–induced cell death, but not vacuolation. These results suggest that c-FLIP_L_ downregulation is a key contributor to CDDO-Me–induced apoptotic cell death, independent of ER-derived vacuolation. Taken together, our results show that ER-derived vacuolation via Ca^2+^ influx and ROS generation as well as caspase activation via c-FLIP_L_ downregulation are responsible for the potent anticancer effects of CDDO-Me on breast cancer cells.

## INTRODUCTION

Breast cancer is one of the most frequently diagnosed cancers, and is a major cause of death among women worldwide [1]. Although the significant morbidity of breast cancer has been somewhat reduced by current treatment modalities, which include surgery, radiotherapy, and adjuvant chemotherapy and/or hormone therapies [2, 3], little progress has been made in treating advanced diseases of the breast. For example, triple-negative breast cancer (TNBC), which lacks receptors for estrogen and progesterone and does not overexpress human epidermal growth factor receptor 2 (HER2), is associated with a significantly higher rate of relapse and lower overall survival rate than other breast cancer subtypes [4]. No specific targeted agents are currently available for the treatment of TNBC and treatment options are limited to cytotoxic chemotherapy [5]. Therefore, there is an urgent need to uncover new breast cancer drugs with acceptable efficacies and toxicities.

Triterpenoids have recently emerged as a unique group of phytochemicals with multifunctional anticancer activities, as demonstrated by promising results in preclinical studies [6, 7]. To improve the anticancer activity of triterpenoids, researchers have synthesized some synthetic triterpenoid derivatives, including cyano-3, 12-dioxoolean-1, 9-dien-28-oic acid (CDDO) and its methyl ester (CDDO-Me) [8–10]. Among them, CDDO-Me potently induces antitumor activity in several types of tumor cells, including leukemia, osteosarcoma, prostate, and lung cancer cells [11–14]. CDDO-Me showed promising anticancer effects and was found to be generally well tolerated in a Phase I trial among patients with advanced solid tumors and lymphomas [15]. The preventive effects of CDDO-Me against breast cancer have been established in several *in vivo* mouse models, including BRCA1-mutated mice [16] and the estrogen receptor-negative mammary carcinogenesis model in polyoma middle T mice [17, 18]. In addition, CDDO-Me has been shown to protect normal breast epithelial cells, but not breast cancer cells, from radiation [19]. However, the cell-death-inducing effects of CDDO-Me on breast cancer and its underlying mechanisms have not been extensively explored. Here, we show for the first time that CDDO-Me induces extensive endoplasmic reticulum (ER)-derived vacuolation prior to cell death in various breast cancer cells. Our results further reveal a reciprocal positive-regulatory loop between Ca^2+^ influx and ROS generation plays a critical role in the CDDO-Me–induced progressive dilation of the ER, contributing to death in these cells. Perturbation of cellular Ca^2+^ and ROS homeostasis by CDDO-Me may lead to accumulation of misfolded proteins in the ER, further aggravating ER stress. Furthermore, we report that CDDO-Me effectively reduced the protein levels of c-FLIP_L_ (cellular FLICE-inhibitory protein), a caspase-8 inhibitor [20], and overexpression of c-FLIP_L_ blocked CDDO-Me–induced cell death, without affecting vacuolation. These results suggest that the CDDO-Me–induced downregulation of c-FLIP_L_ may help tip the balance of breast cancer cells undergoing progressive ER dilation towards caspase-mediated apoptosis. Taken together, our results clearly show that c-FLIP_L_ downregulation and the interplay between Ca^2+^ influx and ROS generation are responsible for the potent anticancer effects of CDDO-Me on breast cancer cells.

## RESULTS

### CDDO-Me exerts potent anti-cancer effects on breast cancer cells

**Figure 1:**
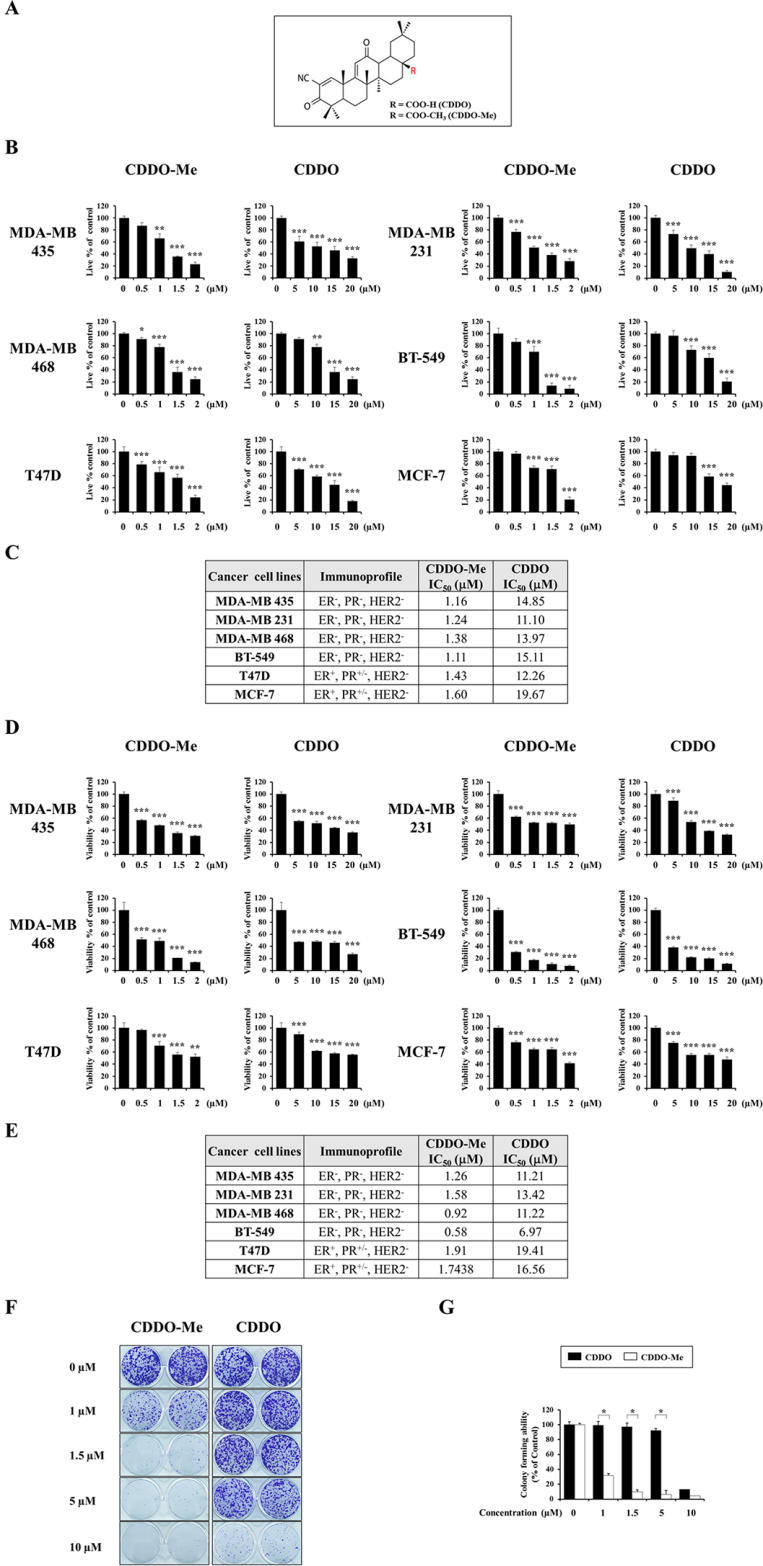
CDDO-Me demonstrates a much stronger anti-cancer effect than CDDO on breast cancer cells **A.** Chemical structures of CDDO and CDDO-Me. **B.** Cells were treated with CDDO or CDDO-Me at the indicated concentrations for 24 h and Live/Dead assay was performed as described in Materials and Methods. Results shown data are mean ± SD of triplicate experiments. **C.** The values of IC_50_ (the concentration of each drug that is required to reduce the viability of treated cells for 24 h to 50%) after the viability assay using calcein-AM and EthD-1 were assessed. **D.** Cells were treated with CDDO or CDDO-Me at the indicated concentrations for 48 h and vability was measured by MTT assay. Results shown data are mean ± SD of triplicate experiments. **E.** The values of IC_50_ (the concentration of each drug that is required to reduce the viability of treated cells for 48 h to 50%) after MTT assay were assessed. **F.** Effects of CDDO-Me and CDDO on the long-term survival of MDA-MB 435 cells. MDA-MB 435 cells seeded on 12 well-plates were treated with CDDO-Me or CDDO at the indicated concentrations for 12 h and then media were replaced with drug-free media. Following the subsequent incubation for 9 days, cells were stained with 0.5% crystal violet. Representative dishes after clonogenic assay are shown. **G.** Colony-forming units were enumerated and expressed as the percentages of control cells. For B and D, statistical significance was determined by one-way ANOVA followed by Bonferroni post hoc tests. *P < 0.05, **P < 0.01, ***P < 0.001 vs. untreated control. For G, statistical significance was determined by unpaired *t*-test. *P < 0.001 between the indicated groups.

To examine the anticancer effects of CDDO and CDDO-Me (Figure 1A) on breast cancer cells, we treated various breast cancer cell lines, including triple-negative breast cancer (TNBC) cells (MDA-MB 435, MDA-MB 231, MDA-MB 468, and BT-549) and non-TNBC cells (T47D and MCF-7) [21–23], with different concentrations of CDDO or CDDO-Me for 24 h, and stained with calcein-AM and EthD-1 to detect live and dead cells, respectively. The percentage of live cells was assessed by counting cells with exclusively green fluorescence, excluding bicolored cells (green and red). Although both CDDO and CDDO-Me concentration-dependently reduced the viability of tested cells (Figure 1B), the 50% inhibitory concentration (IC_50_) values for CDDO-Me toward the respective cancer cell types were ~9–13-fold lower than those of CDDO (Figure 1C). In addition, CDDO-Me demonstrated increased cytotoxicity toward cell types in the TNBC group compared with those in the non-TNBC group. MTT assays performed on cells treated with CDDO-Me or CDDO for 48 h yielded similar results (Figure 1D and 1E). Colony-forming assays also showed that CDDO-Me much more potently inhibited the long-term survival of MDA-MB 435 cells than did CDDO (Figure 1F and 1G). Taken together, these results indicate that CDDO-Me exerts much stronger anticancer effects on breast cancer cells than CDDO.

### CDDO-Me induces progressive, ER-derived vacuolation prior to cell death in breast cancer cells

**Figure 2:**
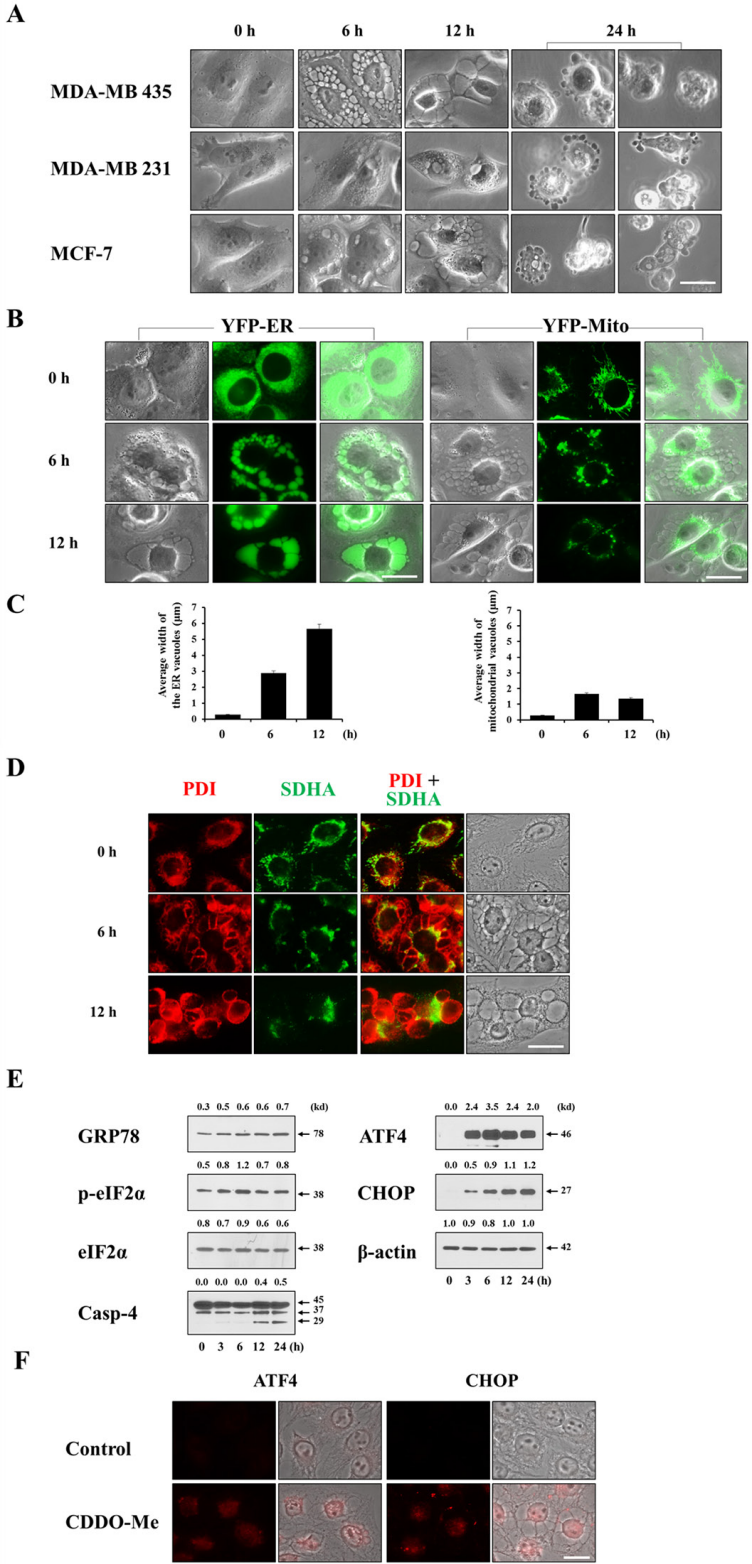
CDDO-Me induces extensive dilation prior to cell death in breast cancer cells **A.** Cells were treated with 1.5 μM CDDO-Me for the indicated time points and observed under the phase contrast microscope. Scale bar: 20 μm. **B.** YFP-ER and YFP-Mito cells were treated with 1.5 μM CDDO-Me for indicated time points and fluorescent microscopy was performed. Scale bar: 20 μm. **C.** The average widths of the vacuoles originated from the ER or mitochondria were measured in YFP-ER or YFP-Mito cells treated with 1.5 μM CDDO-Me for the indicated time points using AxioVision Rel. 4.8 software (Zeiss) as described in Materials and Methods. Marked increase in the width of the ER-derived vacuoles was observed following treatment with 1.5 μM CDDO-Me. **D.** MDA-MB 435 cells were treated with or without 1.5 μM CDDO-Me for indicated time points. Immunocytochemistry using anti-PDI (red) and anti-SDHA (green) antibodies was performed and the representative fluorescence and phase contrast microscopic images of cells are shown. Scale bar: 20 μm **E.** MDA-MB 435 cells were treated with 1.5 μM CDDO-Me for the indicated time points and then Western blotting the proteins associated with ER stress was performed. β-actin was used as a loading control in Western blots. The fold change of protein levels compared to β-actin was determined by a densitometric analysis. **F.** MDA-MB 435 cells were treated with 1.5 μM CDDO-Me for 24 h and immunocytochemistry of ATF4 and CHOP was performed. Scale bar: 20 μm.

Since CDDO-Me demonstrated a much more potent death-inducing effect than CDDO, we focused on the mechanisms underlying CDDO-Me cytotoxicity in breast cancer cells, first examining morphological changes in CDDO-Me–treated cells. Interestingly, we found that a common feature of CDDO-Me treatment in MDA-MB 435, MDA-MB 231 and MCF-7 cells was induction of severe cellular vacuolation prior to cell death (Figure 2A). CDDO-Me–induced cellular vacuolation was also observed in other breast cancer cells, including MDA-MB 468, BT-549, and T47D cells (Supplementary Figure 1). Cellular vacuolation can occur via several pathways involving different cell structures and organelles. Macroautophagy is characterized by sequestration of cytoplasmic components, including damaged organelles, by double-membrane structures called autophagosomes (also called autophagic vacuoles), followed by degradation of the contents of these vacuoles by fusion with the cell's own lysosomes [24]. We first investigated whether CDDO-Me–induced vacuolation and cell death was associated with autophagy by examining cellular responses after knocking down ATG5, Beclin-1 or LAMP2—components of the autophagy process. This examination revealed that neither CDDO-Me–induced cellular vacuolation nor cell death was affected by the knockdown of these proteins (Supplementary Figure 2A–2C). In addition, pretreatment with autophagy inhibitors, including 3-methyladenine (3-MA) and chloroquine (CQ), did not affect CDDO-Me–induced vacuolation or cell death (Supplementary Figure 2D and 2E). Collectively, these results suggest that autophagy is not associated with CDDO-Me–induced cellular responses in these cells. Next, we examined whether vacuoles in CDDO-Me–treated cells originated from the ER and/or mitochondria. For this purpose, we employed MDA-MB 435 sublines stably expressing fluorescence selectively in the ER (YFP-ER cells) or mitochondria (YFP-Mito cells). In untreated YFP-ER cells, the ER appeared as a reticular structure. In contrast, ER fluorescence precisely co-localized with numerous vacuoles in YFP-ER cells treated with 1.5 μM CDDO-Me for 6 h (Figure 2B). After treating with CDDO-Me for 12 h, ER-derived vacuoles were further increased in size, but their numbers were decreased, suggesting the fusion of these ER-derived vacuoles (Figure 2B and 2C). Whereas mitochondria in untreated YFP-Mito cells showed a filamentous and elongated structure, phase-contrast and fluorescence microscopy revealed a fragmented mitochondrial morphology in the cells treated with CDDO-Me for 6 h, or showed co-localization of mitochondrial fluorescence at very small vacuoles around the nuclei, but not at easily discernible vacuoles. After treatment with CDDO-Me for 12 h, most mitochondria appeared to be fragmented in YFP-Mito cells. Immunocytochemical staining for protein disulfide isomerase (PDI), an ER-resident protein, and subunit A of succinate dehydrogenase (SDHA), a mitochondrial protein, showed expression of PDI in a reticulate structure and elongated SDHA expression in untreated MDA-MB 435 cells (Figure 2D)—ER and mitochondrial morphologies similar to those observed in YFP-ER and YFP-Mito cells (Figure 2B). Six hours after treatment with 1.5 μM CDDO-Me, large rings of PDI expression and very small rings of SDHA expression were observed. SDHA-expressing mitochondria-derived vacuoles appeared to be localized near nuclei, whereas PDI-expressing, ER-derived vacuoles were peripheral to the mitochondria-derived vacuoles. After 12 h of CDDO-Me treatment, ER-derived vacuoles were further increased in size, whereas the sizes of mitochondria-derived vacuoles were decreased. These results suggest that CDDO-Me–induced vacuolation may result mainly from dilation of the ER in these breast cancer cells. We next examined whether CDDO-Me induces ER stress, as expected from the severe alterations in the ER structures. Western blotting showed that CDDO-Me treatment induced a marked accumulation of GRP78 (glucose-regulated protein, 78 kDa), phosphorylated eIF2α (eukaryotic translation initiation factor 2-alpha), cleaved caspase-4, ATF4 (activating transcription factor 4) and CHOP (C/EBP homology protein) proteins (Figure 2E)—markers of ER stress. In addition, immunocytochemistry reveled that CDDO-Me markedly upregulated ATF4 and CHOP in nuclei (Figure 2F), indicating that CDDO-Me is an effective ER stress inducer in breast cancer cells.

### CDDO-Me–induced ER-derived vacuolation in breast cancer cells is followed by apoptotic cell death

**Figure 3:**
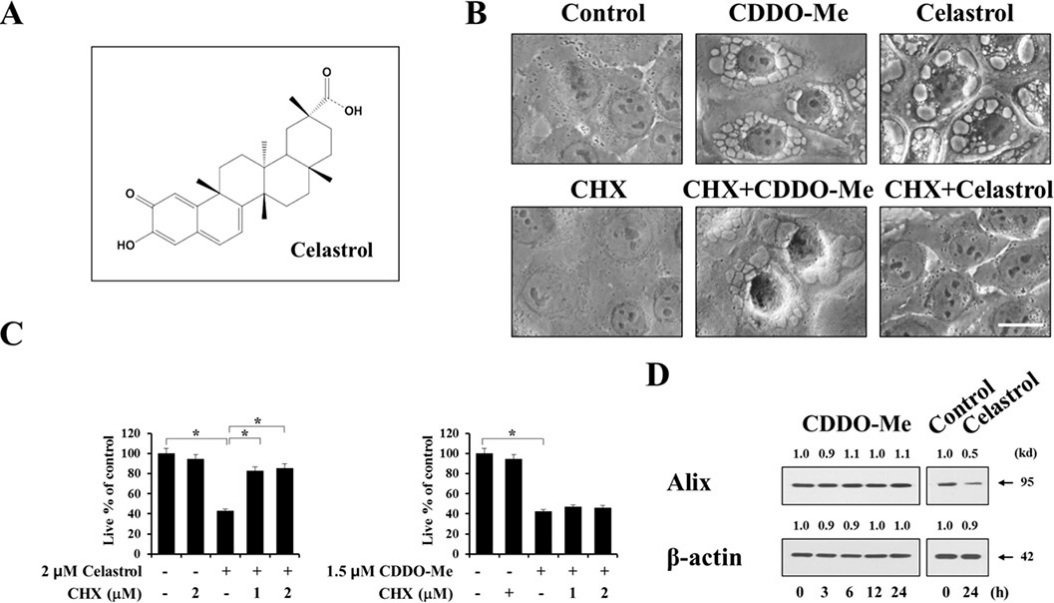
Paraptosis may not be a major cell death mode induced by CDDO-Me in breast cancer cells **A.** Chemical structure of celastrol. **B.** MDA-MB 435 cells were untreated or pretreated with 1 mM cycloheximide and further treated with 1.5 μM CDDO-Me or 2 μM celastrol for 4 h. Cells were observed under the phase contrast microscope. Scale bar: 20 μm. **C.** MDA-MB 435 cells were pretreated with 1 μM cycloheximide and further treated with 1.5 μM CDDO-Me or 2 μM celastrol for 24 h. Viability was assessed using calcein-AM and EthD-1 as described in Materials and Methods. Results shown are mean ± SD of three independent experiments. Statistical significance was determined using one-way ANOVA followed by Bonferroni post hoc tests. *P < 0.001 between the indicated groups. **D.** MDA-MB 435 cells were treated with 1.5 μM CDDO-Me for the indicated time points or treated with 2 μM celastrol for 24 h. Western blot1ting of Alix and β-actin was performed. The fold change of protein levels compared to β-actin was determined by a densitometric analysis.

Previously, a novel non-apoptotic cell death called paraptosis was shown to be accompanied by extensive cellular vacuolation and the origins of paraptotic vacuoles were mitochondria and the ER [25–31]. Since extensive ER dilation preceded cell death in breast cancer cells treated with CDDO-Me, as shown in Figure 2B and 2D, we investigated whether CDDO-Me–treated breast cancer cells die via paraptosis. Recently, we showed that celastrol, a quinone methide triterpenoid (Figure 3A), kills breast and colon cancer cells via paraptosis [31]. Thus, we compared the cellular responses to CDDO-Me with those to celastrol. Although the underlying mechanisms of paraptosis are not clearly understood, *de novo* protein synthesis is known to be required for paraptosis [25, 27, 28]. We first tested the effect of the protein synthesis inhibitor cycloheximide on CDDO-Me–induced cellular responses. We found that cycloheximide pretreatment completely blocked vacuolization in response to celastrol (Figure 3B) and significantly inhibited celastrol-induced cell death (Figure 3C). In contrast, cycloheximide pretreatment had no effect on CDDO-Me–induced vacuolation (Figure 3B) or cell death (Figure 3C). Since we had previously shown that the protein levels of Alix, which is known to inhibit paraptosis [25, 27, 28], are downregulated during paraptosis [28–30], we examined whether CDDO-Me modulated the expression of Alix. We found that Alix protein levels were reduced by celastrol, but not by CDDO-Me (Figure 3D). Collectively, these results suggest that paraptosis may not be a major cell death mode in breast cancer cells treated with CDDO-Me, despite the fact that CDDO-Me initially induces paraptosis-like morphological features.

**Figure 4:**
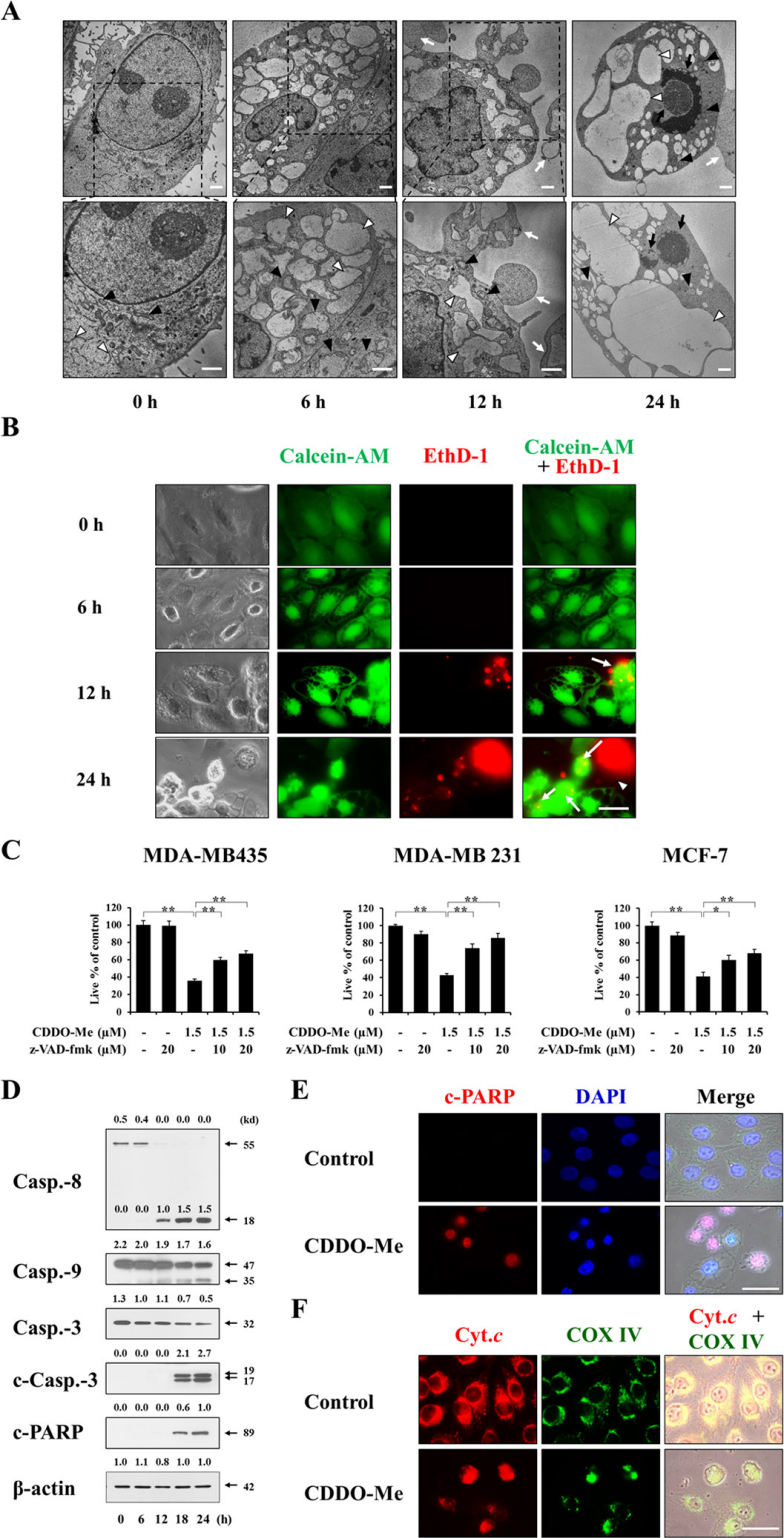
CDDO-Me–induced vacuolation is followed by apoptotic cell death in breast cancer cells **A.** MDA-MB 435 cells were treated with 1.5 μM CDDO-Me for the indicated time points and observed by transmission electron microscopy. White arrow heads and black arrow heads indicate the ER and mitochondria, respectively. White arrows indicate apoptotic bodies and/or blebbing and black arrows denote nuclear condensation and fragmentation. Scale bar: 2 μm. **B.** MDA-MB 435 cells treated with 1.5 μM CDDO-Me for the indicated times were observed by phase-contrast and fluorescence microscopy after staining with calcein-AM and EthD-1. White arrows and white arrowheads indicate bicolored cells and red cells, respectively. Scale bar: 30 μm. **C.** MDA-MB 435, MDA-MB 231, MCF-7 cells were pretreated with the indicated concentrations of z-VAD-fmk for 30 min and further treated with 1.5 μM CDDO-Me for 24 h. Cellular viability was assessed using calcein-AM and EthD-1 as described in Materials and Methods. Results shown are mean ± SD of three independent experiments. Statistical significance was determined by one-way ANOVA followed by Bonferroni post hoc tests. *P < 0.05, **P < 0.01 between the indicated groups. **D.** MDA-MB 435 cells were treated with 1.5 μM for the indicated time points. Whole cell extracts were prepared from the treated cells and subjected to Western blotting. β-actin was used as a loading control in Western blots. The fold change of protein levels compared to β-actin was determined by a densitometric analysis. **E, F.** MDA-MB 435 cells were untreated or treated with 1.5 μM CDDO-Me for 24 h. Immunocytochemistry of the cleaved PARP and staining with DAPI were performed (E). Immunocytochemistry of the cytochrome c (Cyt.c) and the subunit I of cytochrome c oxidase (COX IV) was performed (F) Representative fluorescence microscopic images of cells are shown. Scale bars: 50 μm.

Next, to observe changes in the subcellular structures in CDDO-Me–treated cells undergoing death in more detail, we performed electron microscopy. Untreated MDA-MB 435 cells possessed ER structures with elongated sacs surrounded by a one-layer membrane and mitochondria with intact cristae (Figure 4A). In cells treated with CDDO-Me for 6 h, the intracellular space was occupied by expanded ER structures, and swollen mitochondria were frequently observed. At 12 h post-treatment, ER-derived vacuoles were increased in size but their numbers were decreased, demonstrating fusion of the swollen ER. In contrast, mitochondria were reduced in size at this time, suggesting that cells might undergo mitochondrial fusion earlier during CDDO-Me treatment (6 h) followed by mitochondrial fission. Very interestingly, cytoplasmic blebs and apoptotic bodies containing the dilated ER were frequently observed at 12 h of CDDO-Me treatment. At 24 h, the sizes of ER-derived vacuoles were further enlarged, and chromatin condensation and DNA fragmentation were detected. We next examined how cellular esterase activities (determined using calcein-AM) and plasma membrane integrity (determined using EthD-1) are altered in association with the morphological changes in CDDO-Me-treated MDA-MB 435 cells. Fluorescence microscopy of calcein-AM- and EthD-1-stained cells showed that both untreated cells and many vacuolated cells treated with CDDO-Me were calcein-AM-positive, but were EthD-1-negative if they remained attached to the culture plate (Figure 4B). In contrast, whereas MDA-MB 435 cells undergoing blebbing at 12 or 24 h of CDDOMe treatment appeared bicolored owing to double-staining with calcein-AM and EthD-1, floating cells with shrunken morphologies were calcein-AM-negative and EthD-1-positve. These results suggest that vacuolated cells exhibiting esterase activity might begin to lose their plasma membrane integrity when they undergo blebbing. Once cells lose their ability to adhere to the plate, their plasma membrane integrity is irreversibly lost and they are destined to die. Since CDDO-Me–induced, ER-derived vacuolation in breast cancer cells was followed by the appearance of apoptotic morphologies, we further examined whether CDDO-Me also induced biochemical characteristics of apoptosis, We found that pretreatment with the pan-caspase inhibitor z-VAD-fmk significantly and concentration-dependently inhibited CDDO-Me–induced cell death in MDA-MB 435, MDA-MB 231, and MCF-7 cells (Figure 4C). In addition, Western blotting showed that caspase-8 was very effectively cleaved in MDA-MB 435 cells beginning 12 h after treating with CDDO-Me, and marked processing of caspase-9, csapase-3, and poly-(ADP-ribose) polymerase (PARP) was detected beginning 18 h after CDDO-Me treatment (Figure 4D). Immunocytochemistry showed that PARP cleavage was often detected in CDDO-Me–treated MDA-MB 435 cells containing condensed and/or fragmented nuclei (Figure 4E). Furthermore, immunocytochemical staining for COX IV, a mitochondrial protein, and cytochrome c revealed that cytochrome c was released from mitochondria into the cytosol in CDDO-Me–treated cells (Figure 4F). Taken together, these results show that CDDO-Me initially induces paraptosis-like morphological features in breast cancer cells through ER-derived vacuolation, but ultimately leads to caspase-mediated apoptotic cell death.

### Ca^2+^ influx is crucial for CDDO-Me–induced vacuolation and subsequent apoptotic cell death

**Figure 5:**
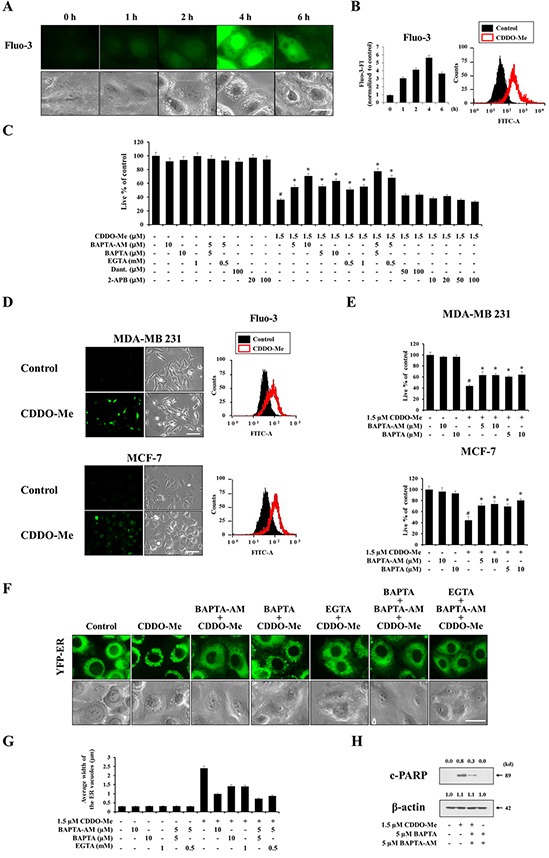
Ca^2+^ influx is critical for CDDO-Me–induced vacuolation and subsequent apoptotic cell death **A, B.** MDA-MB 435 cells treated with 1.5 μM CDDO-Me for the indicated time points were stained with 2.5 μM Fluo-3. Cells were observed under the fluorescence and the phase contrast microscope (A). Cells were processed for FACS analysis. Fluo-3 fluorescence intensities (FI) in cells treated with 1.5 μM CDDO-Me were compared with that of untreated cells and denoted in the graph (B *left*). Histogram for the cells treated with 1.5 μM CDDO-Me for 4 h is shown (B *right*). X axis, fluorescence intensity, Y axis, relative number of cells. **C.** MDA-MB 435 cells were untreated or pretreated with the indicated Ca^2+^ antagonists at the indicated concentrations for 30 min and further treated with 1.5 μM CDDO-Me for 24 h. Cellular viability was assessed using calcein-AM and EthD-1 as described in Matereials and Methods. **D.** MDA-MB 231 or MCF-7 cells untreated or treated with 1.5 μM CDDO-Me for 3 h, 6 h, respectively, were stained with 2.5 μM Fluo-3 and observed under the fluorescence microscope (*left*) or processed for FACS analysis (*right*). Scale bar: 50 μm. **E.** MDA-MB 231 and MCF-7 cells were untreated or pretreated with the indicated Ca^2+^ antagonists for 30 min and further treated with 1.5 μM CDDO-Me for 24 h. Cellular viability was assessed using calcein-AM and EthD-1. **F.** YFP-ER cells were pretreated with 10 μM BAPTA, 10 μM BAPTA-AM or 1 mM EGTA alone for 30 min and further treated with 1.5 μM CDDO-Me for 6 h. Or cells were pretreated with 5 μM BAPTA plus 5 μM BAPTA-AM or 0.5 mM EGTA plus 5 μM BAPTA-AM for 30 min and further treated with 1.5 μM CDDO-Me for 6 h. Cells were observed by fluorescence and phase contrast microscopy. Scale bar: 20 μm. **G.** The changes in the widths of the ER-derived vacuoles were quantitatively measured using AxioVision Rel. 4.8 software, as described in Materials and Methods section. **H.** MDA-MB 435 cells were untreated or treated with BAPTA plus BAPTA-AM at the indicated concentrations and further treated with 1.5 μM CDDO-Me for 24 h. Cell extract were prepared for Western blotting of cleaved PARP and β-actin. For C and E, statistical significance was determined by one-way ANOVA followed by Bonferroni post hoc tests. #*P* < 0.001 vs. untreated control; *P < 0.001 vs. CDDO-Me treatment. Results shown are mean ± SD of triplicate experiments.

Since the ER is a major reservoir of intracellular Ca^2+^, and CDDO-Me severely altered ER structures via dilation, we next tested whether CDDO-Me perturbed intracellular Ca^2+^ homeostasis. Fluorescence microscopy and flow cytometry using the cell-permeable Ca^2+^-indicator dye Fluo-3 demonstrated that treatment of MDA-MB 435 cells with 1.5 μM CDDO-Me dramatically increased intracellular Ca^2+^ levels, which peaked at 4 h post-treatment (Figure 5A and 5B). An examination of the functional significance of this increase in Ca^2+^ levels showed that BAPTA-AM, a cell-permeable acetoxymethyl ester of the Ca^2+^ scavenger BAPTA, concentration-dependently inhibited CDDO-Me–induced cell death in MDA-MB 435 cells (Figure 5C), suggesting that the increase in intracellular Ca^2+^ levels is critically associated with this cell death. Next, we investigated the sources of increased Ca^2+^ following CDDO-Me treatment. Intracellular Ca^2+^ levels can be increased either by influx of Ca^2+^ from the extracellular milieu or release from the ER via two major Ca^2+^ release receptors: the IP_3_ receptor (IP_3_R) and ryanodine receptor (RyR) [32, 33]. We found that two scavengers of extracellular Ca^2+^, BAPTA and EGTA, concentration-dependently inhibited CDDO-Me–induced cell death. Furthermore, inclusion of the intracellular Ca^2+^ scavenger BAPTA-AM further enhanced this cytoprotective effect compared to treatment with BAPTA or EGTA alone (Figure 5C). In contrast, pretreatment with dantrolene, a specific inhibitor of the RyR, or 2-APB, a relatively selective inhibitor of IP_3_Rs, had no effect on CDDO-Me–induced cell death. These results suggest that the influx of extracellular Ca^2+^ rather than Ca^2+^ released from the ER is the primary contributor to this cell death. CDDO-Me treatment also increased Ca^2+^ levels in MDA-MB 231 and MCF-7 cells (Figure 5D) and pretreatment with BAPTA or BAPTA-AM inhibited CDDO-Me–induced cell death in these breast cancer cells (Figure 5E). Next, we used YFP-ER cells to examine whether scavenging of intra- and/or extracellular Ca^2+^ affected CDDO-Me–induced dilation of the ER. We found that scavenging of intracellular Ca^2+^ using BAPTA-AM or scavenging of extracellular Ca^2+^ using BAPTA or EGTA significantly inhibited CDDO-Me–induced ER dilation in MDA-MB 435 cells (Figure 5F and 5G). These inhibitory effects of BAPTA and EGTA were further enhanced by inclusion of BAPTA-AM during pretreatment. Notably, BAPTA plus BAPTA-AM markedly inhibited CDDO-Me–induced cleavage of PARP (Figure 5H). Taken together, these results suggest that an increase in Ca^2+^ levels mediated by influx of extracellular Ca^2+^ triggers ER dilation and contributes to apoptotic cell death in breast cancer cells.

### H_2_O_2_–associated ROS generation critically contributes to CDDO-Me–induced vacuolation and cell death

**Figure 6:**
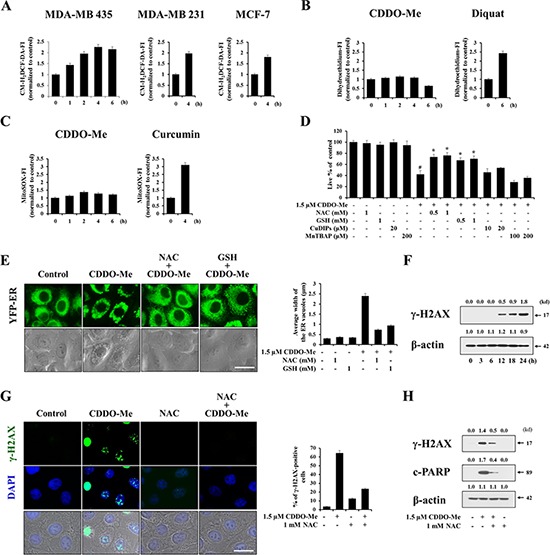
Generation of H_2_O_2_ critically contributes to CDDO-Me–induced vacuolation and cell death **A–C.** Cells were treated with 1.5 μM CDDO-Me for the indicated time points. Treated cells were exposed to 5 μM CM-H_2_DCF-DA (A), 20 μM dihydroethidium (B) or 2.5 μM Mito-SOX (C) for 30 min and analyzed by flow cytometry. Fluorescence intensities (FI) of the respective dye were assessed and H_2_O_2_, superoxide, mitochondrial superoxide levels were compared between cells treated with and without CDDO-Me for the indicated durations. The fold changes of FI the respective dye are shown in the graph. As the positive inducer of superoxide or mitochondrial superoxide, diquat or curcumin was used. **D.** MDA-MB 435 cells were pretreated with the indicated concentrations of various antioxidants at the indicated concentrations for 30 min and further treated with 1.5 μM CDDO-Me for 24 h. Cellular viability was assessed using calcein-AM and EthD-1. Results shown are mean ± SD of triplicate experiments. Statistical significance was determined by one-way ANOVA followed by Bonferroni post hoc tests. #*P* < 0.01 vs. untreated control; *P < 0.01, compared to CDDO-Me treatment. **E.** YFP-ER cells were pretreated with 1 mM NAC or 1 mM GSH and further treated with 1.5 μM CDDO-Me for 6 h, and then observed under the fluorescence and phase contrast microscope (*left*). The changes in the widths of the ER-derived vacuoles were quantitatively measured as described in Materials and Methods (*right*). Scale bar: 30 μm. **F.** MDA-MB 435 cells were treated with 1.5 μM CDDO-Me for the indicated time points. Western blotting of γ-H2AX and β-actin was performed. **G.** MDA-MB 435 cells were pretreated with 1 mM NAC and further treated with 1.5 μM CDDO-Me for 24 h. *Left*: Cells were fixed and immunostained for γ-H2AX; nuclei were counterstained with DAPI. *Right*: Percentages of cells with more than 30 γ-H2AX foci per cells were assessed and presented graphically. Scale bar: 30 μm. **H.** MDA-MB 435 cells were pretreated with NAC and further treated with 1.5 μM CDDO-Me for 24 h. Cell extracts were prepared for Western blotting of γ-H2AX, cleaved PARP and β-actin.

It has previously been shown that ROS are involved in CDDO-Me–induced growth inhibition and apoptosis in several types of cancer cells [34–36]. Thus, we next examined whether CDDO-Me also generates ROS in breast cancer cells and, if so, whether this contributes to the ER-derived vacuolation and subsequent apoptosis. For this purpose, we measured the generation of H_2_O_2_-associated ROS, superoxide, or mitochondrial superoxide using 5-(and-6)-chloromethyl-2′, 7′-dichlorodihydrofluorescein diacetate, acetyl ester (CM-H_2_DCF-DA), dihydroethidium, or MitoSOX-Red, respectively, following CDDO-Me treatment. CM-H_2_DCF-DA increases when it is oxidized by H_2_O_2_ and free radicals downstream of H_2_O_2_ [37]. Dihydroethidium is known to form a red fluorescent product (ethidium) which intercalates with DNA [38]. MitoSOX-Red is relatively insensitive to oxidation by H_2_O_2_ but is sensitive to oxidation by superoxide (O_2_^.−^) [37]. Flow cytometry using CM-H_2_DCF-DA showed that treatment MDA-MB 435 cells with 1.5 μM CDDO-Me increased H_2_O_2_ levels, which peaked at 4 h post-treatment (Figure 6A), a time course that paralleled changes in Ca^2+^ levels (see Figure 5A and 5B). Similar responses were obtained in MDA-MB 231 and MCF-7 cells (Figure 6A). In contrast, measurements using dihydroethidium revealed that CDDO-Me very marginally increased superoxide levels compared with diquat [39], used as a positive control for the induction of superoxide (Figure 6B). In addition, Mito-SOX staining revealed a very slight increase in mitochondrial superoxide levels compared with curcumin, used as a positive control for mitochondrial superoxide induction [28] (Figure 6C). Taken together, these results suggest that H_2_O_2_ is the major ROS induced by CDDO-Me. A further investigation of the functional significance of ROS in CDDO-Me–induced cell death using various antioxidants revealed that, whereas pretreatment of MDA-MB 435 cells with NAC or GSH (general antioxidants) very effectively inhibited CDDO-Me–induced cell death, pretreatment with MnTBAP (a MnSOD mimetic) or CuDIPS (a Cu/ZnSOD mimetic) did not significantly affect it (Figure 6D). Furthermore, pretreatment with NAC or GSH also very effectively blocked CDDO-Me–induced ER dilation in YFP-ER cells (Figure 6E). Collectively, these results indicate that H_2_O_2_-associated ROS critically contribute to ER-derived vacuolation and subsequent apoptotic cell death. Next, we examined whether increases in ROS caused by CDDO-Me might induce DNA damage. Western blot analyses revealed that CDDO-Me treatment progressively increased the protein levels of γ-H2AX (Figure 6F). In addition, we found that CDDO-Me increased the percentage of γ-H2AX-positive cells, an effect that was blocked by NAC pretreatment (Figure 6G and 6H). NAC pretreatment also markedly blocked CDDO-Me–induced increases in γ-H2AX protein levels as well as levels of the cleaved form of PARP (Figure 6H). Therefore, these results indicate that DNA damage caused by increased ROS may also contribute to CDDO-Me–induced cell death.

### A reciprocal positive-regulatory relationship exists among Ca^2+^ influx, ROS generation, and accumulation of misfolded proteins in CDDO- Me–induced vacuolation and subsequent cell death

**Figure 7:**
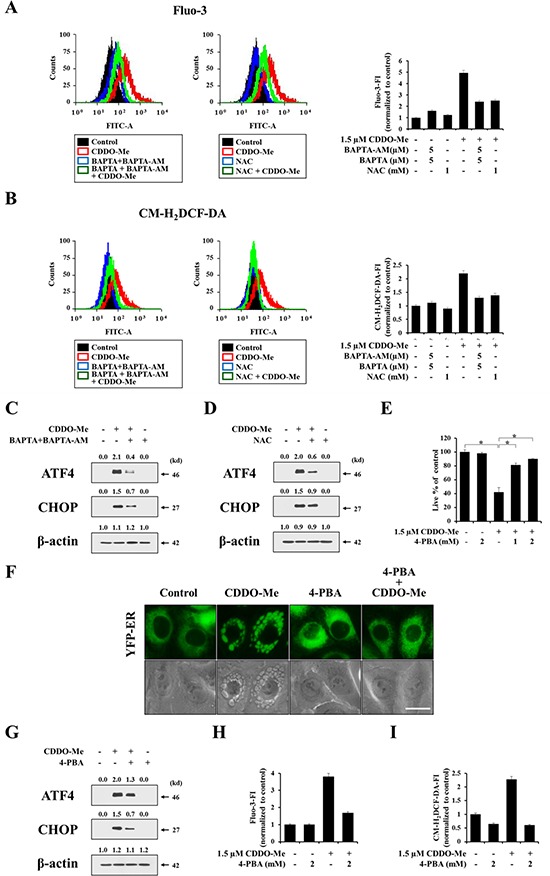
CDDO-Me–induced Ca^2+^ influx, ROS generation, protein misfolding modulate each other, critically contributing to vacuolation and subsequent apoptosis **A, B.** MDA-MB 435 cells were pretreated with 5 μM BAPTA-AM plus 5 μM BAPTA or 1 mM NAC and further treated with or without 1.5 μM CDDO-Me for 4 h. Cells were exposed to 2.5 μM Fluo-3 for 20 min (A) or 5 μM CM-H_2_DCF-DA and for 30 min (B) and analyzed by flow cytometry. Histograms for the cells treated as indicated are shown (*left*). X axis, fluorescence intensity, Y axis, relative number of cells. The fold changes of Fluo-3 FI (A) or CM-H_2_DCF-DA FI (B) compared with that of untreated cells are shown in the graph (*right*). **C, D.** MDA-MB 435 cells were pretreated with 5 μM BAPTA plus 5 μM BAPTA-AM (C) or 1 mM NAC (D), and further treated with 1.5 μM CDDO-Me for 6 h. Western blotting of the indicated proteins was performed. **E.** MDA-MB 435 cells were pretreated with 4-PBA and further treated with 1.5 μM CDDO-Me for 24 h. Cell viability was assessed using calcein-AM and EthD-1. Results shown are mean ± SD (*n* = 5). Statistical significance was determined by one-way ANOVA followed by Bonferroni post hoc tests. *P < 0.01 between the indicated groups. **F.** YFP-ER cells were pretreated with 2 mM 4-PBA and further treated with 1.5 μM CDDO-Me for 6 h, and then observed under the fluorescence and phase-contrast microscope. Scale bar: 20 μm. **G.** MDA-MB 435 cells were pretreated with 4-PBA and further treated with 1.5 μM CDDO-Me for 6 h and Western blotting of ATF4, CHOP and β-actin was performed. **H, I.** MDA-MB 435 cells were pretreated with 2 mM 4-PBA and further treated with or without 1.5 μM CDDO-Me for 4 h. (H) Cells were exposed to 2.5 μM Fluo-3 for 20 min and analyzed by flow cytometry. The fold changes of Fluo-3 FI compared with that of untreated cells are shown in the graph. (I) Cells were exposed to 5 μM CM-H_2_DCF-DA and for 30 min and analyzed by flow cytometry. The fold changes of CM-H_2_DCF-DA FI compared with that of untreated cells are shown in the graph.

Since both intracellular Ca^2+^ and ROS were increased by CDDO-Me and peaked over a similar time frame, as shown in Figure 5B and 6A, we further investigated the relationship between Ca^2+^ and ROS. Flow cytometry revealed that CDDO-Me–induced increases in Ca^2+^ levels and ROS generation were both effectively blocked not only by BAPTA plus BAPTA-AM but also by NAC pretreatment (Figure 7A and 7B). Thus, these results indicate that a positive interplay between Ca^2+^ and ROS is involved in CDDO-Me–induced vacuolation and subsequent apoptotic cell death. We next investigated whether scavenging of Ca^2+^ or ROS affected CDDO-Me–induced ER stress. Pretreatment of MDA-MB 435 cells with BAPTA plus BAPTA-AM or NAC effectively inhibited CDDO-Me–induced upregulation of ATF4 and CHOP (Figure 7C and 7D), suggesting that perturbation of Ca^2+^ and ROS homeostasis may trigger ER stress, leading to ER-derived vacuolation and cell death. It is known that ER stress can be initiated by abnormal accumulation of proteins, and 4-phenylbutyrate (4-PBA), a chemical chaperone, has been shown to alleviate ER stress-mediated cell damage [40, 41]. Therefore, we next investigated whether attenuation of ER stress using 4-PBA affected CDDO-Me–induced cellular responses. We found that pretreatment with 4-PBA very effectively blocked CDDO-Me–induced cell death as well as vacuolation (Figure 7E and 7F), and attenuated CDDO-Me–induced upregulation of CHOP and ATF4 (Figure 7G). These results suggest that 4-PBA might act through its chaperone property to enhance the refolding of unfolded or misfolded proteins. Finally, we examined the effect of 4-PBA on the increase in Ca^2+^ and H_2_O_2_ induced by CDDO-Me. Very interestingly, 4-PBA pretreatment dramatically reduced the increase in Ca^2+^ and ROS levels, assessed by flow cytometry using Fluo-3 and CM-H_2_DCF-DA, respectively (Figure 7H and 7I). Taken together, these results indicate that there exists a complex reciprocal interplay among Ca^2+^, ROS, and protein misfolding in CDDO-Me–induced ER vacuolation and cell death. Increases in Ca^2+^ and ROS levels induced by CDDO-Me may trigger protein misfolding in the ER, leading to ER stress and ER dilation. In addition, structural changes in the ER may further contribute to the perturbation of cellular Ca^2+^ and ROS homeostasis, creating a vicious cycle that results in cell death.

### c-FLIP_L_ downregulation plays a critical role in CDDO-Me–induced apoptotic cell death, but not in ER-derived vacuolation

**Figure 8:**
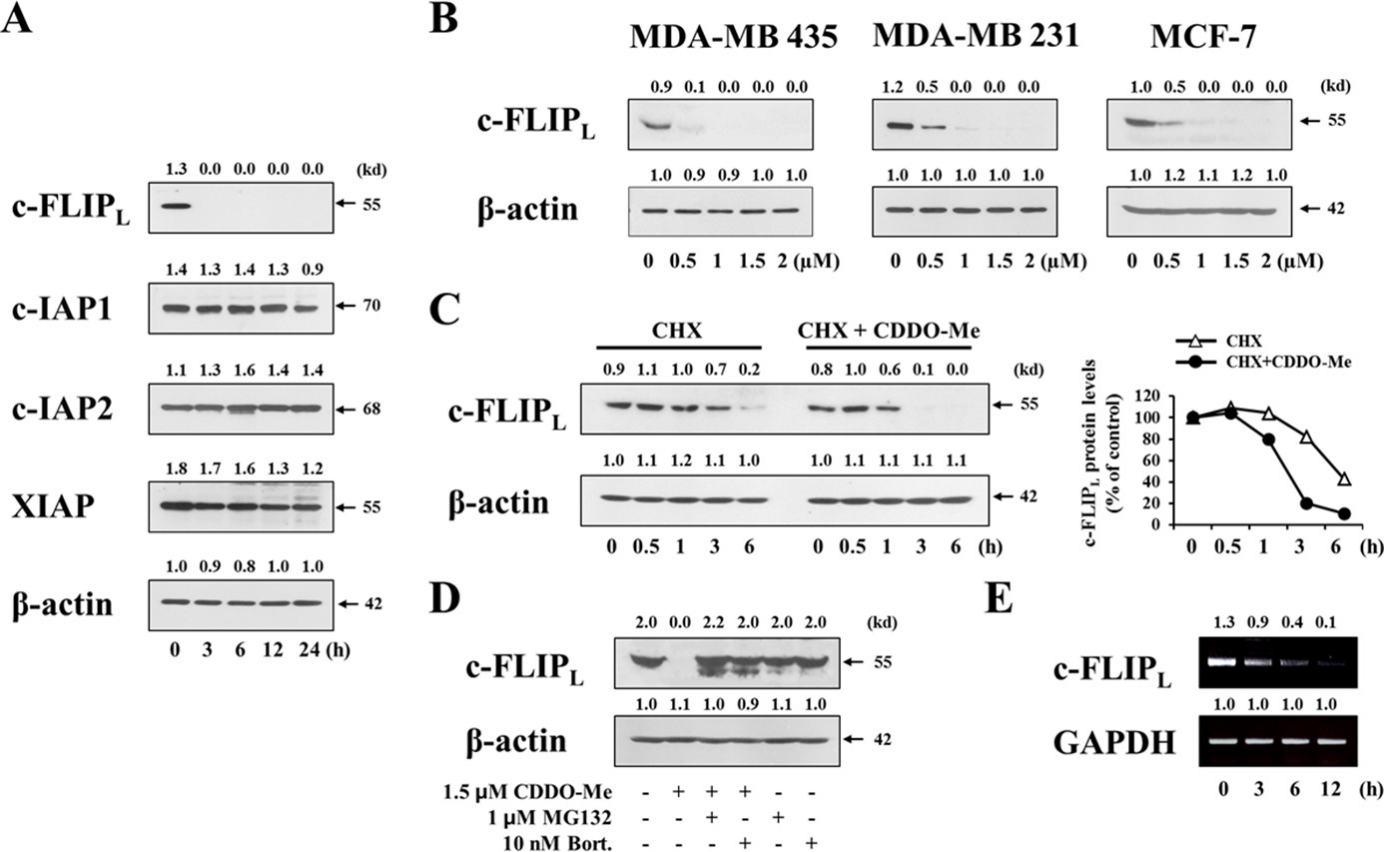
CDDO-Me downregulates in breast cancer cells both transcriptionally and post-translationally **A.** MDA-MB 435 cells were treated with 1.5 μM CDDO-Me for the indicated time points and then Western blotting of the indicated proteins was performed. β-actin was used as a loading control in Western blots. The fold change of protein levels compared to β-actin was determined by a densitometric analysis. **B.** Cells were treated with the indicated doses of CDDO-Me for 24 h and then Western blotting of c-FLIP_L_ was performed. β-actin was used as a loading control in Western blots. **C.** MDA-MB 435 cells were treated with or without 1.5 μM CDDO-Me in the presence of 1 μM CHX for the indicated time periods and Western blotting of c-FLIP_L_ and β-actin was performed. The fold change of c-FLIP_L_ protein levels compared to β-actin was determined by a densitometric analysis. **D.** MDA-MB 435cells were pretreated with 1 μM MG132 or 10 nM bortezomib and further treated with 1.5 μM CDDO-Me for 24 h. Western blotting of c-FLIP_L_ and β-actin was performed. **E.** MDA-MB 435 cells were treated with 1.5 μM CDDO-Me for the indicated time points. c-FLIP_L_ mRNA levels were determined by RT-PCR. The level of GAPDH was used as loading controls. The fold change of c-FLIP_L_ mRNA levels compared to GAPDH was determined by a densitometric analysis.

To clarify the possible contribution of other factor(s) to CDDO-Me–induced, caspase-dependent apoptotic cell death, we examined whether CDDO-Me modulates the expression of cellular caspase antagonists. Among the tested inhibitor of apoptosis proteins (IAPs), c-IAP2 protein levels were not altered by treatment with 1.5 μM CDDO-Me treatment, although XIAP and c-IAP1 protein levels were slightly reduced (Figure 8A). Interestingly, the protein levels of c-FLIP_L_, a caspase-8 inhibitor [20], were strikingly reduced beginning 3 h after treating with CDDO-Me; c-FLIP_S_ proteins were not detected in these cells in the absence or presence of CDDO-Me. In addition, CDDO-Me treatment concentration-dependently reduced the protein levels of c-FLIP_L_ in MDA-MB 435, MDA-MB 231, and MCF-7 cells (Figure 8B). To determine whether these effects reflected a CDDO-Me–induced decrease in c-FLIP_L_ protein stability, we measured c-FLIP_L_ protein levels by Western blotting after blocking protein synthesis with cycloheximide. In the absence of CDDO-Me (cycloheximide alone), c-FLIP_L_ protein levels began to decrease within 3 h; in contrast, CDDO-Me decreased c-FLIP_L_ protein levels within 1 h in cycloheximide-treated cells (Figure 8C). These results indicate that CDDO-Me reduces the protein stability of c-FLIP_L_ in these cells. Since it has been shown that c-FLIP protein levels are regulated by proteasome activity [42], we further tested the effect of proteasome inhibitors on CDDO-Me–induced decreases in c-FLIP_L_ protein levels. We found that pretreatment with either MG132 or bortezomib effectively reversed the reduction in c-FLIP_L_ protein levels induced by CDDO-Me (Figure 8D), suggesting that CDDO-Me–induced downregulation of c-FLIP_L_ protein is attributable to proteasome-mediated degradation. Interestingly, RT-PCR analyses showed that CDDO-Me also progressively downregulated c-FLIP_L_ mRNA levels (Figure 8E). Taken together, these results suggest that CDDO-Me potently downregulates c-FLIP_L_ both at the transcriptional and post-transcriptional control.

**Figure 9:**
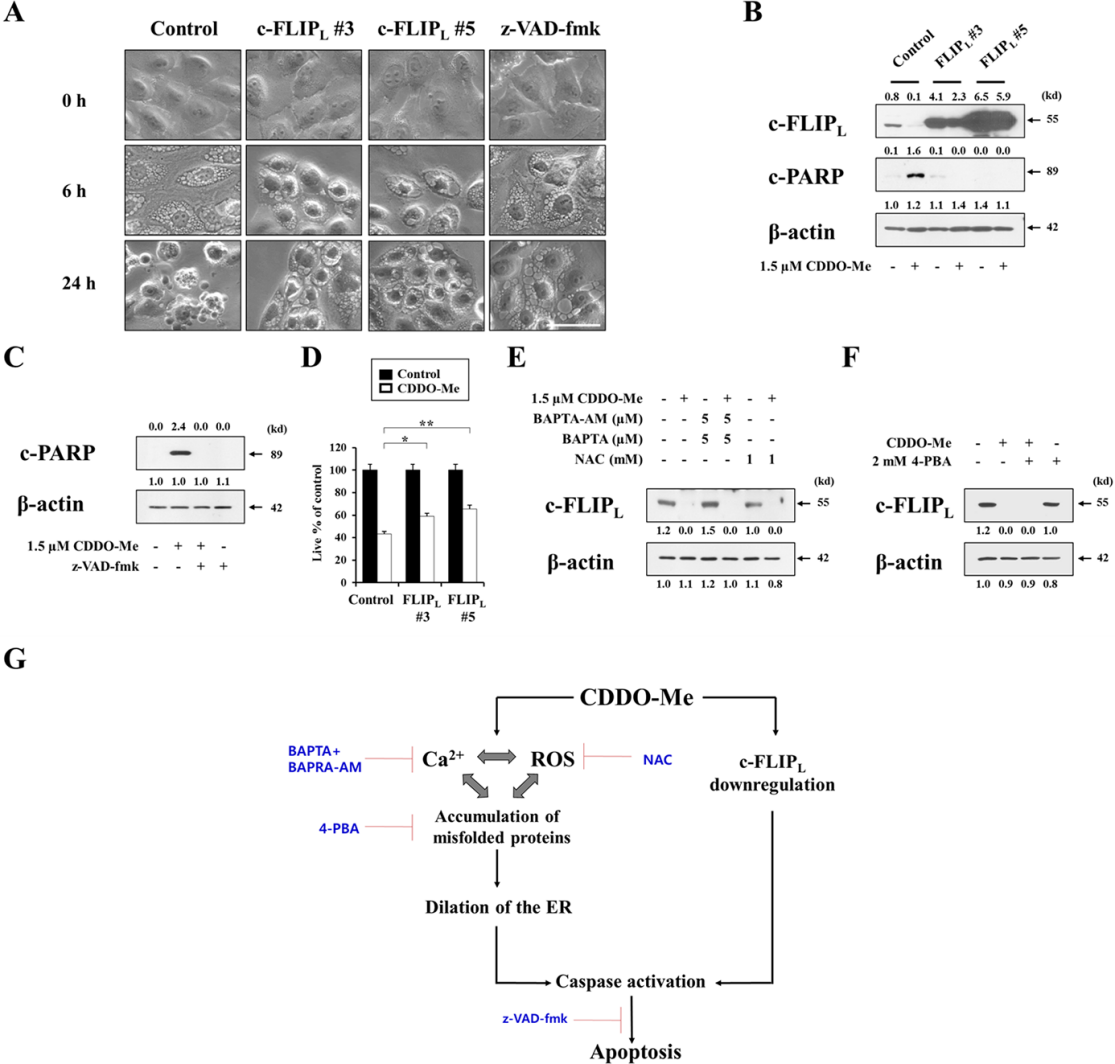
c-FLIPL downregulation is critical for CDDO-Me–induced apoptotic cell death, but not for vacuolation **A.** Control cells (MDA-MB 435/Vector) and two independent MDA-MB 435 sublines overexpressing c-FLIP_L_ (FLIP_L_#3 and FLIP_L_#5) were treated with 1.5 μM CDDO-Me for the indicated time points. Control cells were pretreated with 20 μM z-VAD-fmk and further treated with 1.5 μM CDDO-Me for the indicated time points. Cells were observed under the phase contrast microscope. Scale bars: 50 μm. **B.** Control cells (MDA-MB 435/Vector) and two independent c-FLIP_L_–overexpressing cells were treated with 1.5 μM CDDO-Me for 24 h. The protein levels of c-FLIP_L_ and cleaved PARP were examined by Western blotting in the control cells and c-FLIP_L_-overexpressing cells. β-actin was used as a loading control in Western blots. **C.** MDA-MB 435 cells were untreated or pretreated with 20 μM z-VAD-fmk and further treated with 1.5 μM CDDO-Me for 24 h. Western blotting of c-FLIP_L_ and cleaved PARP was performed. **D.** Control cells and two c-FLIP_L_–overexpressing cells were treated with 1.5 μM CDDO-Me for 24 h the indicated time points. Cellular viability was measured using calcein-AM and EthD-1. Results shown are mean ± SD. Statistical significance was calculated using Student's *t*-test. *P < 0.05, **P < 0.001 between the indicated groups. **E.** MDA-MB 435 cells were pretreated with NAC or BAPTA plus BAPTA-AM and further treated with 1.5 μM CDDO-Me for 24 h. Western blotting of c-FLIP_L_ and β-actin was performed. **F.** MDA-MB 435 cells were pretreated with 2 mM 4-PBA and further treated with 1.5 μM CDDO-Me for 24 h. Expression of c-FLIP_L_ and β-actin was detected by Western blotting. **G.** Schematic model for CDDO-Me–induced cell death in breast cancer cells. CDDO-Me–induced Ca^2+^ influx, ROS generation, and protein misfolding trigger ER-derived vacuolation and subsequent apoptotic cell death. In addition, CDDO-Me-induced c-FLIP_L_ downregulation contributes to caspase-mediated apoptotic cell death, independent of the dilation of the ER.

To investigate the functional significance of c-FLIP_L_ downregulation in CDDO-Me–induced cellular responses, we first tested whether exogenously expressed c-FLIP_L_ could block CDDO-Me–induced vacuolation. To this end, we established MDA-MB 435 sublines stably overexpressing c-FLIP_L_ and observed the cellular morphologies following treatment of these cells with CDDO-Me. Interestingly, c-FLIP_L_ overexpression did not block CDDO-Me–induced vacuolation, which was observed beginning 6 h after treating with CDDO-Me (Figure 9A). However, c-FLIP_L_ overexpression did inhibit CDDO-Me–induced cellular shrinkage, blebbing, and formation of apoptotic bodies, morphological changes that were observed in control cells after treatment with CDDO-Me for 24 h. Similarly, z-VAD-fmk pretreatment also failed to block CDDO-Me–induced vacuolation, but did inhibit CDDO-Me–induced apoptotic morphologies. In addition, c-FLIP_L_ overexpression markedly blocked CDDO-Me–induced cleavage of PARP (Figure 9B), effects similar to those of z-VAD-fmk (Figure 9C). When we quantitatively assessed the effect of c-FLIP_L_ overexpression on CDDO-Me–induced cell death, c-FLIP_L_ overexpression partially, but significantly, restored cellular viability in response to CDDO-Me (Figure 9D). These results suggest that c-FLIP_L_ downregulation is critically involved in CDDO-Me–induced caspase-mediated apoptotic cell death. A further examination of the effect of Ca^2+^ and ROS on c-FLIP_L_ downregulation showed that scavenging Ca^2+^ with BAPTA plus BAPTA-AM or ROS using NAC failed to block CDDO-Me–induced c-FLIP_L_ downregulation (Figure 9E). 4-PBA pretreatment also did not affect CDDO-Me–induced c-FLIP_L_ downregulation (Figure 9F). These results suggest that CDDO-Me–induced c-FLIP_L_ downregulation is independent of ER-derived vacuolation, which is mainly induced by increases in intracellular Ca^2+^ and ROS as well as protein misfolding. However, CDDO-Me–induced c-FLIP_L_ downregulation may help tip the balance towards apoptotic cell death in breast cancer cells with structural and functional defects in the ER. Collectively, our results clearly show that ER dilation via Ca^2+^ influx and ROS generation as well as c–FLIP_L_ downregulation are critically involved in the potent anticancer effects of CDDO-Me on malignant breast cancer cells (Figure 9G).

## DISCUSSION

The plant-derived triterpenoids have been used medicinally in many Asian countries because of their anti-inflammatory and antioxidant properties [43]. A large number of triterpenoids exhibit cytotoxicity against a variety of cancer cells, and cancer preventive, as well as anticancer efficacy in preclinical animal models [6, 7, 44]. To improve the anticancer activity of triterpenoids, researchers have synthesized some synthetic triterpenoid derivatives, including CDDO and its methyl ester, CDDO-Me [8–10]. Among them, CDDO-Me seems promising with a good safety profile in human clinical trials [15, 45, 46]. As the targets of CDDO-Me, nuclear factor-kappa B NF-κB [47], Akt/protein kinase B1/mammalian target of rapamycin [48], JAK/STAT (Janus-activated kinase/signal transducer and activator of transcription) [49], the death receptor-induced extrinsic apoptotic pathway [14, 50], and telomerase [13] have been proposed. However, the molecular mechanisms underlying the anticancer effects of CDDO-Me remain incompletely understood. In addition, although the preventive activity of CDDO-Me against breast cancer has been shown in several *in vivo* mouse models [16–18], its cell-death-inducing activity and therapeutic potential against breast cancer have not yet been extensively explored. We show here that CDDO-Me is much more cytotoxic toward various breast cancer cells than CDDO, requiring ~10-fold lower concentrations to produce similar cytotoxicity and eliminating the clonogenicity of MDA-MB 435 cells at much lower concentrations. In addition, CDDO-Me was more cytotoxic toward TNBC cells compared with non-TNBC cells. A further investigation of the mechanisms underlying CDDO-Me–induced cell death revealed that CDDO-Me treatment caused morphological changes in common in various breast cancer cells, inducing extensive vacuolation prior to cell death. Recently, we showed that curcumin, dimethoxycurcumin, and celastrol kill malignant breast cancer cells primarily by inducing paraptosis [28–31]. Paraptosis is a cell death mode characterized by: extensive cytoplasmic vacuolization that arises via swelling of the ER and mitochondria [25–31]; the lack of characteristic apoptotic features, such as pyknosis, DNA fragmentation and caspase activation [25, 26, 28–31]; insensitivity to caspase inhibitors [25, 27–31]. In contrast, paraptosis is inhibited by blocking protein synthesis [25, 27–31] and Alix is negatively involved in it [25, 28–30]. However, the following evidence indicates that paraptosis may not be a major contributor to CDDO-Me–induced cell death in breast cancer cells: 1) CDDO-Me–induced vacuoles were mainly derived from the ER, whereas mitochondria may be fragmented after transient fusion; 2) CDDO-Me–induced vacuolation and subsequent cell death were not inhibitable by the protein synthesis inhibitor cycloheximide; and 3) Alix was not downregulated by CDDO-Me. In addition, mitochondrial superoxide levels, which we found critically contribute to paraptosis induced by curcumin [28] or celastrol [31], were not markedly increased by CDDO-Me, and CDDO-Me–induced cell death was not affected by pretreatment with the MnSOD-mimetic, MnTBAP. Taken together, these results suggest that CDDO-Me does not kill breast cancer cells through activation of paraptosis, despite initially inducing paraptosis-like morphological features. Interestingly, CDDO-Me–induced vacuolation was followed by typical apoptotic morphologies, including cellular shrinkage, cytoplasmic blebbing, formation of apoptotic bodies and chromatin condensation. In addition, CDDO-Me–induced cell death was accompanied by the release of mitochondrial cytochrome c and depended on caspases. Collectively, these results indicate that, following CDDO-Me treatment, severely vacuolated breast cancer cells ultimately die through the activation of apoptotic machineries.

We found that the extensive ER vacuolization induced by CDDO-Me results in ER stress, as evidenced by upregulation of GRP78, phosphorylation of eIF2α, cleavage of caspase-4, and nuclear translocation of ATF4 and CHOP. Interestingly, we found that ER-derived vacuolation was closely linked to the marked increases in intracellular Ca^2+^ levels induced by CDDO-Me. Scavenging of intracellular Ca^2+^ using BAPTA-AM significantly rescued MDA-MB 435 cells from CDDO-Me–induced ER-derived vacuolation and subsequent apoptosis, demonstrating the functional importance of these increases in intracellular Ca^2+^ levels. We further found that vacuolation and cell death were effectively inhibited by the extracellular Ca^2+^-chelators, BAPTA and EGTA, but not by inhibitors of IP_3_Rs (2-APB) or RyRs (dantrolene), suggesting that extracellular Ca^2+^ entry is critical for these cellular responses to CDDO-Me. In this context, we considered the possible involvement of L-type voltage-gated Ca^2+^ channels or TRPV1 (transient receptor potential vanilloid 1) channels, which have been shown to mediate the Ca^2+^ influx responsible for glutamate-induced apoptosis in VSC 4.1 cell line (a motor neuron and neuroblastoma hybrid cell line) [51] and capsaicin-induced apoptosis in glioma cells [52], respectively. However, we found that various L-type channel antagonists (verapamil, lercanidipine, and amlodipine) and the TRPV antagonist, capsazepine, had no effect on CDDO-Me–induced vacuolation and cell death (Supplementary Figure 3). We did find that pretreatment with the non-selective Ca^2+^ channel blocker bepridil [53] or T-type Ca^2+^ channel blocker NiCl_2_ [54] very effectively blocked CDDO-Me–induced vacuolation and cell death (Supplementary Figure 4), suggesting that CDDO-Me may trigger Ca^2+^ influx via T-type Ca^2+^ channels. However, further detailed studies are required to precisely identify the responsible Ca^2+^ channels and to clarify how Ca^2+^ influx leads to the ER dilation that contributes to CDDO-Me–induced apoptotic cell death.

Interestingly, we found that CDDO-Me treatment increased both intracellular Ca^2+^ and ROS levels, which peaked at a similar time (~4 h) after treatment. Pretreatment with antioxidants (NAC and GSH), like pretreatment with Ca^2+^ scavengers, very effectively inhibited CDDO-Me–induced vacuolation and cell death, demonstrating the critical involvement of ROS in these processes. Consistent with our results, CDDO-Me–induced apoptosis in ovarian, pancreatic and colon cancer cells was reported to depend on ROS generation [34–36], although the relationship between ROS generation and CDDO-Me–induced vacuolation was not addressed in these previous studies. Interestingly, we observed that antioxidants effectively blocked not only ROS generation but also the increase in intracellular Ca^2+^ levels induced by CDDO-Me. Moreover, Ca^2+^ chelation with BAPTA plus BAPTA-AM also inhibited increases in ROS and Ca^2+^ levels, suggesting that Ca^2+^ influx and ROS generation modulate each other, triggering CDDO-Me–induced vacuolation and cell death. Consistent with our results, an influx of extracellular Ca^2+^ was shown to critically contribute to the vulnerability of neurons to glutamate [55]. In this process, ROS generation was followed by Ca^2+^ influx. Additionally, glutamate-induced cytotoxicity in mouse hippocampal HT22 cells was reported to be associated with ROS generation, and treatment with EGTA or the calcium channel blocker, CoCl_2_, attenuated ROS generation [56]. Furthermore, during tumor necrosis factor (TNF)-α- and glutamate-induced retinal ganglion cell death, both ROS levels and intracellular Ca^2+^ influx are increased [57]. In their study, PRDX6 (peroxiredoxin 6) overexpression was shown to protect against this cell death by reducing ROS levels and limiting increases in Ca^2+^ influx. These observations suggest that Ca^2+^ influx and ROS generation are also closely linked with each other in these cell death models. In addition, we found that the role of increased Ca^2+^ and ROS levels in ER-derived vacuolation and cell death is closely linked to ER stress, because scavenging of Ca^2+^ or ROS effectively inhibited CDDO-Me–induced upregulation of ATF4 and CHOP. The chemical chaperone 4-PBA also potently inhibited CDDO-Me–induced vacuolation and cell death, suggesting the involvement of ER stress due to protein misfolding in this response. Very interestingly, 4-PBA pretreatment markedly reduced the CDDO-Me–induced increase in Ca^2+^ and ROS levels. These results suggest that the existence of a complex reciprocal modulatory relationship among Ca^2+^, ROS, and protein misfolding that leads to ER-derived vacuolation and cell death. Since protein folding in the ER is exquisitely sensitive to changes in the environment, such as altered Ca^2+^ levels and oxidative conditions, CDDO-Me–induced Ca^2+^ influx and ROS generation may trigger accumulation of misfolded proteins in the ER, ultimately contributing to irreversible structural and functional impairment of the ER. However, further study is required to clarify whether depletion or overload of Ca^2+^ in the ER is critical for this ER stress.

The fact that CDDO-Me induces caspase-mediated apoptosis despite morphological similarities to paraptosis invites speculation about the underlying mechanism. Upregulation of c-FLIP has been found in various tumor types, and its silencing has been shown to restore apoptotic responses to cytokines and various chemotherapeutic agents [58]. In our study, treatment with CDDO-Me, even at a low concentration, consistently reduced c-FLIP_L_ protein levels in breast cancer cells while very effectively promoting caspase processing, in particular that of caspase-8. Overexpression of c-FLIP_L_ significantly inhibited CDDO-Me–induced cell death and very effectively blocked the cleavage of PARP, effects similar to those of z-VAD-fmk, suggesting that c-FLIP_L_ downregulation plays a critical role in triggering caspase-mediated apoptotic cell death. However interestingly, neither c-FLIP_L_ overexpression nor z-VAD-fmk pretreatment markedly affected CDDO-Me–induced vacuolation. In addition, whereas scavenging of Ca^2+^ or ROS as well as 4-PBA pretreatment significantly inhibited CDDO-Me–induced vacuolation and cell death, neither affected c-FLIP_L_ downregulation, indicating that c-FLIP_L_ downregulation is not directly related to Ca^2+^ influx or ROS generation during CDDO-Me-induced cell death. Thus, we presume that CDDO-Me–induced downregulation of c-FLIP_L_, a caspase-8 inhibitor [20], may help tip the balance towards caspase-mediated apoptotic cell death in breast cancer cells undergoing progressive ER dilation.

In sum, we show here that ER-derived vacuolation via Ca^2+^ influx, ROS generation, and protein misfolding as well as caspase activation via c-FLIP_L_ downregulation is responsible for the potent anticancer effects of CDDO-Me on breast cancer cells. Thus, CDDO-Me may be a promising anticancer agent in the treatment of breast cancer.

## MATERIALS AND METHODS

### Chemicals and antibodies

CDDO-Me and CDDO were purchased from Cayman Chemical. N-acetylcysteine (NAC), reduced glutathione (GSH), ethylene glycol tetraacetic acid (EGTA), 1, 2-bis(o-aminophenoxy)ethane-N, N, N'N’-tetraacetic acid (BAPTA), 1, 2-bis(o-aminophenoxy)ethane-N, N, N'N’-tetraacetic acid acetoxymethyl ester (BAPTA-AM), sodium 4-phenylbutyrate (4-PBA), 3-(4, 5-dimethylthiazol-2-yl)-2, 5-diphenyltetrazplium bromide (MTT) were purchased from Sigma-Aldrich. Rhod-2-AM, Fluo-3-AM, 5-(and-6)-chloromethyl-2′, 7′-dichlorodihydrofluorescein diacetate, acetyl ester (CM-H_2_DCF-DA), dihydroethidine (DHE), MitoSOX-Red, calcein- acetoxymethyl ester (calcein-AM), and ethidium homodimer-1 (EthD-1) were from Molecular Probes. 2-aminoethosxydiphenyl borate (2-APB) and Mn (III) tetrakis-(4-benzoic acid)-porphyrin chloride (MnTBAP) were obtained from Calbiochem. Dantrolene was obtained from Alexis Biochemicals. Copper bis-3, 5-diisopropylsalicylate (CuDIPs) was purchased from Abcam. The following antibodies were used: anti-β-actin, anti-cleaved PARP, anti-γ-H2AX (Abcam); anti-caspase-4, anti-XIAP (Assay designs), anti-ATF4, anti-c-IAP1, anti-c-IAP2 (Santa Cruz Biotechnologies); anti-caspase-8, anti-caspase-3 (Stressgen); anti-caspase-9 (Novus Biologicals); anti-phospho-eIF2α, anti-total eIF2α, anti-cleaved caspase-3, anti-Alix, anti-GRP78, anti-CHOP (Cell Signaling); anti-c-FLIP (Enzo Life Sciences); HRP-conjugated anti-rabbit IgG and HRP-conjugated anti-mouse IgG (Molecular Probes).

### Cell culture

The MDA-MB 435, MDA-MB 231, MDA-MB 468, BT-549, T47D and MCF-7 human breast cancer cells were purchased from American Type Culture Collection. MDA-MB 435, MDA-MB 231, MDA-MB 468, MCF-7 cells were cultured in DMEM supplemented with 10% fetal bovine serum (FBS) and antibiotics (GIBCO-BRL) and BT-549, T47D cells were cultured in RPMI supplemented with 10% FBS and antibiotics. Cells incubated in 5% CO_2_ at 37°C.

### Examination of the changes in the ER and mitochondria employing the stable cell lines expressing the fluorescence specifically in mitochondria or endoplasmic reticulum

To establish the stable cell lines expressing the fluorescence specifically in mitochondria or endoplasmic reticulum (ER), MDA-MB 435 cells were transfected with the *pEYFP-ER* or *pEYFP-Mito* vector (Clontech). The *pEYFP-ER* vector encodes a fusion protein consisting of EYFP, flanked on the 5′-end by the ER-targeting sequence of the luminal-resident protein calreticulin [59, 60], and on the 3′-end by a conserved KDEL motif present in luminal ER proteins [61]. *pEYFP-Mito* encodes a fusion of EYFP and the mitochondrial targeting sequence from subunit VIII of human cytochrome c oxidase [62], which is localized at the mitochondrial inner membrane. Therefore, the fluorescence derived from the *pEYFP-ER* vector is expressed in the ER lumen and the fluorescence derived from the *pEYFP-Mito* vector is expressed in the inner mitochondrial membrane. Stable cell lines expressing *pEYFP-ER* or *pEYFP-Mito* (YFP-ER or YFP-Mito) were selected with fresh medium containing 500 μg/mL G418 (Calbiochem). To quantitatively measure the dilation of the ER and mitochondria induced by CDDO-Me, we analyzed the average width of the vacuoles originated from the ER and mitochondria in YFP-ER cells and YFP-Mito cells using AxioVision Rel. 4.8 software (Zeiss). More than 200 clearly identifiable vacuoles derived from the ER in 50 YFP-ER cells and more than 200 clearly identifiable vacuoles derived from mitochondria in 50 YFP-Mito cells per experiment, randomly selected, were measured in three independent experiments.

### Determination of cellular viability using calcein-AM and EthD-1 (Live/Dead assay)

Cells (5 × 10^4^ cells) were cultured in 24-well plates and treated as indicated. For measurement of cellular viability, 2 μmol/L calcein-AM, a green fluorescent indicator of the intracellular esterase activity of cells, and 4 μmol/L EthD-1, a red fluorescent indicator of membrane-damaged (dead) cells, were added to each well, and the plates were incubated for 5 min in 5% CO_2_ at 37°C. Cells were then observed under a fluorescence microscope (Axiovert 200M; Carl Zeiss) equipped with Zeiss filter sets #46 and #64HE. Viable cells, corresponding to those that exclusively exhibited green fluorescence, were counted in five fields per well at 200 × magnification. Only exclusively green cells were counted as live because bicolored (green and red) cells cannot be unambiguously assigned to live or dead groups. The percentage of live cells (Live %), calculated as green cells/(green + red + bicolored cells), was normalized to that of untreated control cells (100%).

### Determination of cellular viability by an MTT assay

Cells were plated in 96-well plates at a concentration of 1 × 10^4^ cells/ml. After treatments, MTT assay was performed according to the manufacturer's protocol (Sigma). Absorption at 570 nm was normalized to that of untreated control (100%), and the results were expressed as Viability % of control.

### Western blotting

Western blotting was performed as described in our previous studies [30]. The representative results from at least three independent experiments are shown. The respective protein band intensity was quantified by densitometric analysis using the NIH ImageJ program.

### Immunocytochemistry

After treatments, cells were fixed with acetone/methanol (1:1) for 5 min at −20 °C and blocking in 5% BSA in PBS for 30 min. Fixed cells were incubated overnight at 4°C with primary antibody [anti-SDHA (1:500, mouse, Invitrogen), anti-PDI (1:500, rabbit, Stressgen), anti-ATF4 (1:500, rabbit, Santa Cruz Biotechnologies), anti-CHOP (1:500, rabbit, Cell Signaling), anti-cytochrome c (1:500, mouse, BD Transduction Lab.), or anti-COX IV (1:500, mouse, GeneTex)]diluted in PBS and then washed three times in PBS and incubated for 1 h at room temperature with anti-rabbit or anti-mouse Alexa Fluor 488 or 594 (1:1000, Molecular Probes). Slides were mounted with ProLong Gold antifade mounting reagent (Molecular probes) and cell staining was visualized with a fluorescence microscope using Zeiss filter sets #46 and #64HE.

### Measurement of reactive oxygen species (ROS) and mitochondrial superoxide production

CM-H_2_DCF-DA, dihydroethidine (DHE) and Mito-SOX fluorescent probes were used to measure the intracellular generation of hydrogen peroxide (H_2_O_2_), superoxide anions (O^2·−^) and mitochondrial superoxide, respectively. Briefly, 3 × 10^5^ MDA-MB 435 cells were plated in 6-well plates and allowed to attach overnight. Cells were incubated with or without CDDO-Me and then incubated with 5 μM of H_2_DCF-DA for 30 min, 20 μM of DHE for 30 min, or 2.5 μM MitoSOX-Red for 20 min in the dark at 37°C. After washing with Hank's Buffered Salt Solution (HBSS) containing Ca^2+^ and Mg^2+^, cells were further processed for fluorescence-activated cell sorting (FACS) analysis using a FACScan system (BD Biosciences). Data were analyzed using WinMDI 2.8 software (BD Biosciences).

### Measurement of cytosolic and mitochondrial Ca^2+^ levels

To measure [Ca^2+^]_c_, treated cells were incubated with 2.5 μM Fluo-3-AM at 37°C for 20 min, washed with HBSS (without Ca^2+^ or Mg^2+^), and analyzed immediately by flow cytometry or fluorescence microscopy.

### Transmission electron microscopy

Cells were prefixed in Karnovsky's solution (1% paraformaldehyde, 2% glutaraldehyde, 2 mM calcium chloride, 0.1 M cacodylate buffer, pH 7.4) for 2 h and washed with cacodylate buffer. Post-fixing was carried out in 1% osmium tetroxide and 1.5% potassium ferrocyanide for 1 h. After dehydration with 50–100% alcohol, the cells were embedded in Poly/Bed 812 resin (Pelco, Redding, CA), polymerized, and observed under electron microscope (EM 902A, Zeiss).

### Statistical analysis

All values were presented as mean ± SD from at least three separate experiments. Statistical significances of differences were determined using Student's *t*-test or one-way ANOVA followed by a Bonferroni multiple comparison test, as indicated in the figure legend. A *P*-value < 0.05 was considered significant.

## SUPPLEMENTARY FIGURES


