# Supplementary material for: Ca2+ influx-mediated dilation of the endoplasmic reticulum and c-FLIPL downregulation trigger CDDO-Me–induced apoptosis in breast cancer cells
Source: Oncotarget. 2015 May 25;6(25):21173–92. doi: 10.18632/oncotarget.4065 (PMC4673258; doi:10.18632/oncotarget.4065)
Supplement: Supplementary file 1 [file oncotarget-06-21173-s001.pdf]

SUPPLEMENTARY FIGURES

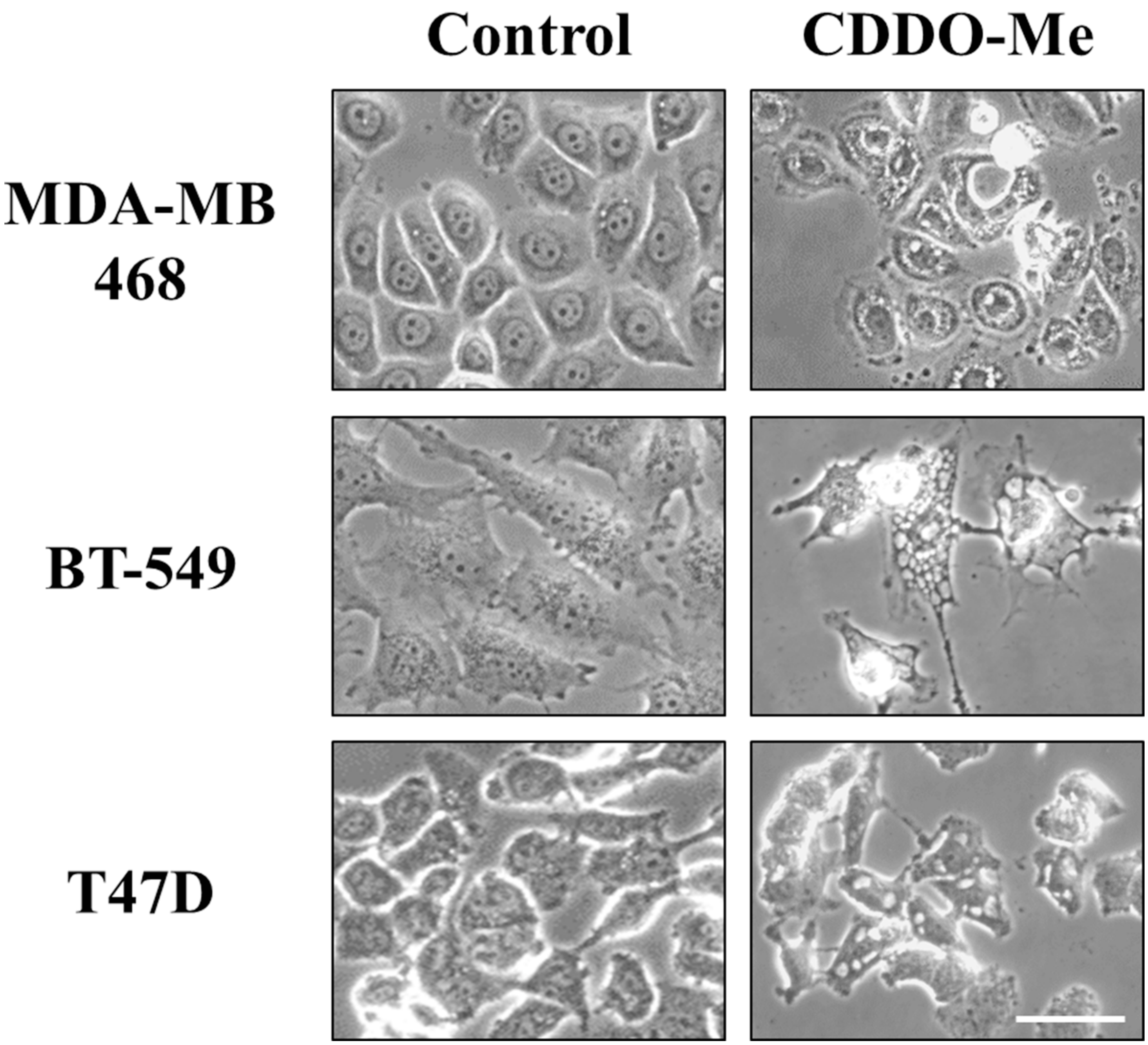

Supplementary Figure S1: CDDO-Me–induced cellular vacuolation in breast cancer cells. Cells were treated with 1.5 μM CDDO-Me for 12 h and observed under the phase contrast microscope. Scale bar: 50 μm.

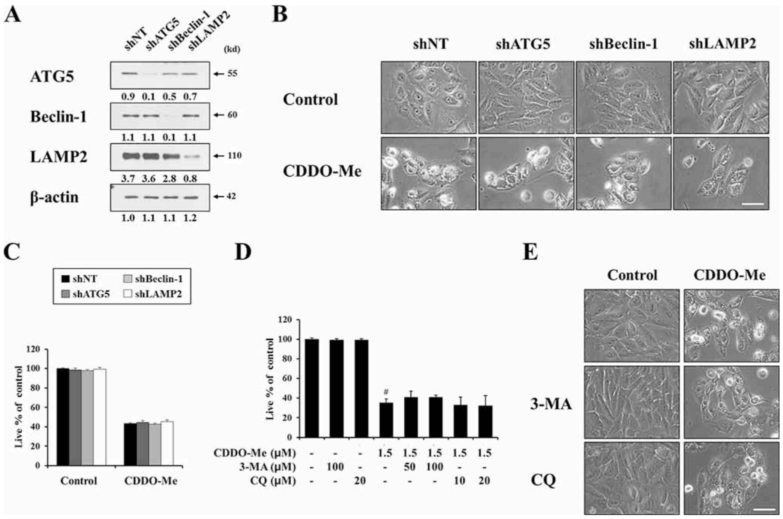

Supplementary Figure S2: Autophagy is not associated with CDDO-Me–induced vacuolation and subsequent cell death. A. MDA-MB 435 cells were treated with the lentivirus encoding the control non-targeting RNA (shNT), ATG5, Beclin-1 or LAMP2 shRNA and their knockdown was confirmed by Western blotting. The fold change of protein levels compared to shNT was determined by a densitometric analysis. **B, C.** MDA-MB 435 cells treated with the lentivirus encoding the control non-targeting RNA (shNT), ATG5, Beclin-1 or LAMP2 shRNA were further treated with or without 1.5 µM CDDO-Me for 24 h. Cells were observed under a phase contrast microscope. Scale bar: 50 µm (B). Cellular viability was assessed using calcein-AM and EthD-1 (C). **D.** MDA-MB 435 cells were pretreated with the indicated concentrations of 3-MA or chloroquine for 30 min and further treated with 1.5 µM CDDO-Me for 24 h. Cellular viability was assessed using calcein-AM and EthD-1. **E.** MDA-MB 435 cells were pretreated with 100 µM 3-MA or 20 µM CQ for 30 min and further treated with 1.5 µM CDDO-Me for 24 h. Cells were observed under a phase contrast microscope. Scale bar: 50 µm. For C and D, statistical significance was determined using one-way ANOVA followed by Bonferroni post hoc tests. #*P* < 0.01 vs. untreated control. Results shown are mean ± SD of triplicate experiments.

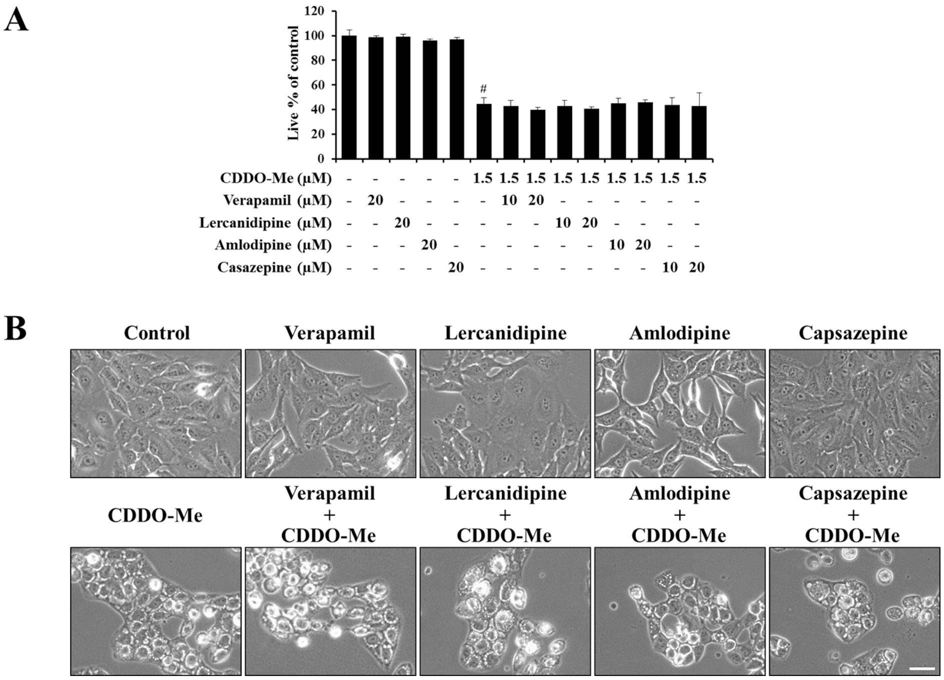

Supplementary Figure S3: Effects of several Ca^{2+} channel blockers on CDDO-Me–induced vacuolation and cell death. A. MDA-MB 435 cells were pretreated with the indicated concentrations of verapamil, lercanidipine, and amlodipine, capsazepine for 30 min and further treated with or without 1.5 µM CDDO-Me for 24 h. Cellular viability was assessed using calcein-AM and EthD-1. Results shown are mean ± SD of triplicate experiments. Statistical significance was determined using one-way ANOVA followed by Bonferroni post hoc tests. #*P* < 0.01 vs. untreated control. **B.** MDA-MB 435 cells were treated with 20 µM verapamil, 20 µM lercanidipine, 20 µM amlodipine, or 20 µM capsazepine and further treated with or without 1.5 µM CDDO-Me for 24 h observed under a phase contrast microscope. Scale bar: 50 µm.

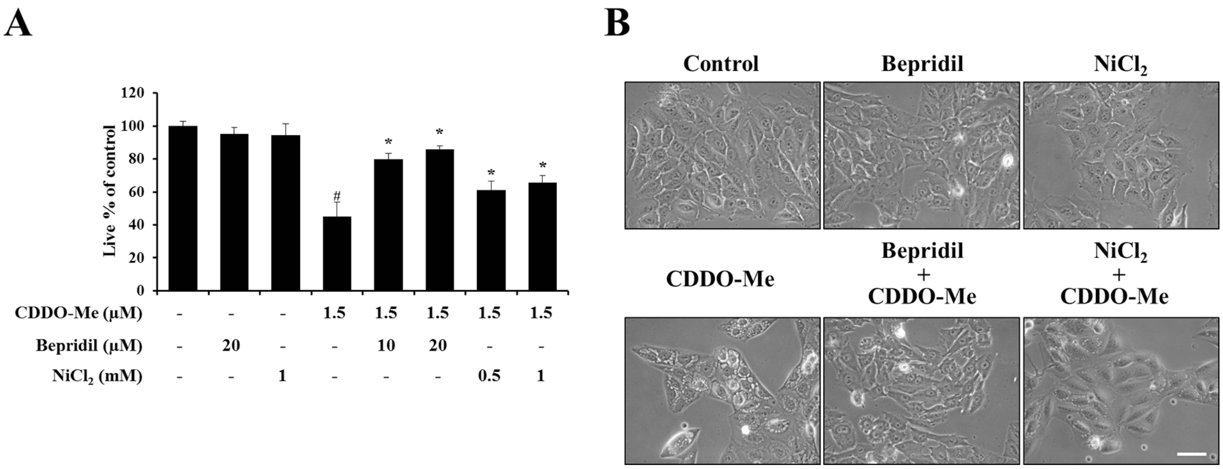

Supplementary Figure S4: Effects of bepridil or $NiCl_2$ on CDDO-Me–induced vacuolation and cell death. A. MDA-MB 435 cells were pretreated with the indicated concentrations of bepridil or $NiCl_2$ for 30 min and further treated with 1.5 μM CDDO-Me for 24 h. Cellular viability was assessed using calcein-AM and EthD-1. Results shown are mean ± SD of triplicate experiments. Statistical significance was determined using one-way ANOVA followed by Bonferroni post hoc tests. #$P < 0.01$ vs. untreated control; *$P < 0.01$ vs. CDDO-Me treatment. **B.** MDA-MB 435 cells were pretreated with 20 μM bepridil or 1 mM $NiCl_2$ for 30 min and further treated with or without 1.5 μM CDDO-Me for 12 h. Cells were observed under a phase contrast microscope. Scale bar: 50 μm.
